# The expectations we build: reframing chronic pain outcomes as a systems level public health issue

**DOI:** 10.3389/fpubh.2026.1919984

**Published:** 2026-09-21

**Authors:** LaDarius Woods

**Affiliations:** Department of Physical Therapy, School of Health Professions, University of Alabama at Birmingham, Birmingham, AL, United States

**Keywords:** chronic pain, health equity, health professions education, pain literacy, public health, systems level care

## Abstract

Chronic pain is a public health issue that affects a substantial proportion of adults and limits life, work, and social participation. Although pain is understood as a multidimensional experience shaped by biological, psychological, social, and contextual factors, chronic pain care is often organized around the expectation that pain can be identified, treated, and resolved through targeted clinical intervention. This perspective argues that chronic pain outcomes are often misattributed to individual factors such as adherence, motivation, or resilience, when they may also reflect expectations generated by healthcare systems, health professions education, payment structures, and broader societal messaging. These systems can reinforce expectations of fixability, rapid resolution, and passive care, making it harder to support recovery, resilience, and sustained engagement. When those expectations are not met, individuals with chronic pain may be labeled as noncompliant, resistant, or insufficiently resilient, shifting attention away from the structures that shaped both the expectations and the outcomes. This mismatch is particularly consequential for underserved populations, who may experience higher pain burden, reduced access to comprehensive care, and greater structural barriers to sustained engagement. Reframing chronic pain as a systems level public health issue highlights the need to align clinical care, public education, professional training, and prevention oriented policies with the complex realities of chronic pain. Improving chronic pain outcomes requires systems that create realistic expectations, support resilience and recovery, and reduce the misattribution of system shaped outcomes to individual failure.

## 1 Introduction

Chronic pain is among the most consequential public health problems in the United States. In 2023, 24.3% of U. S. adults reported chronic pain, and 8.5% reported pain that limited life or work activities (1). Its effects extend beyond symptoms to function, participation, and work activities (1). This public health burden also requires greater attention to prevention, education, recovery, and long term adaptation.

This perspective argues that chronic pain outcomes are often misattributed to individual factors such as adherence, motivation, or resilience, when they may also reflect expectations created by healthcare systems, health professions education, payment structures, and societal messaging. These expectations position pain as identifiable, fixable, and rapidly resolved. When chronic pain does not follow this path, attention may shift toward perceived patient/client failure rather than the systems that helped create the expectation. Reframing outcomes as system influenced does not remove individual agency. It places behavior within the conditions, messages, resources, and constraints that shape how people understand and respond to pain.

Disability scholarship offers one useful lens for this argument without assuming that all people with chronic pain identify as disabled. Medical models tend to locate disability within individual pathology and emphasize treatment or cure, whereas social and critical disability perspectives draw attention to how bodily experience interacts with social environments, ableism, and institutional structures (2, 3). Applied to chronic pain, this distinction is incomplete because pain can itself be disabling even as systems amplify its effects. This Perspective is informed by disability scholarship and draws selectively on concepts from structural competency, epistemic injustice, and structural violence to illuminate specific aspects of how expectations, credibility, access, and recovery are shaped across levels (4–6).

## 2 How systems build expectations about chronic pain

Chronic pain care is often shaped by an expectation of fixability: that pain can be located, explained, treated, and resolved through targeted intervention. Patient/clients seek relief, clarity, and a plan, while clinicians are trained to identify sources of symptoms and intervene. Yet chronic pain may persist beyond expected tissue healing, fluctuate, and reflect biological, psychological, social, and contextual factors (7, 8). When systems communicate that pain should be quickly identifiable and correctable, ongoing pain may be interpreted as treatment or patient/client failure rather than a predictable challenge of chronic pain care.

Healthcare systems also construct these expectations. In the U. S., pain care is often organized around encounters after pain has emerged, worsened, or become disabling. The National Pain Strategy identified prevention and care, disparities, service delivery and payment, professional education and training, and public education and communication as priorities (9). Yet systems still emphasize evaluation, diagnosis, imaging, medication, procedures, or referral after symptoms become disruptive, reinforcing pain as a problem to locate and correct rather than one shaped across individual, interpersonal, community, and systems levels.

Diagnostic and interventional care can shape treatment expectations. Imaging, medications, injections, and surgical consultations may be appropriate, but their prominence can imply that pain should correspond to a structural problem or require definitive intervention (10, 11). Low back pain guidelines discourage routine imaging for nonspecific low back pain and recommend it when severe or progressive neurologic deficits or serious underlying conditions are suspected (10). Calls to reduce low value care likewise emphasize evidence, patient/client centered practice, and population level approaches (11). When structural explanations are easier to access than pain education or prevention focused care, active long term strategies can appear secondary.

Health professions education also shapes pain expectations. Across disciplines, learners are often trained to evaluate symptoms, identify pathology, and select interventions. These skills are essential, but they can support a narrow view when chronic pain is framed mainly as a problem of tissue, structure, or disease (2, 3). Evidence from medical education suggests that pain education has often been insufficient relative to the prevalence and public health impact of pain (12). Structural competency offers one response by training clinicians to recognize how policies, institutions, and other upstream structures shape what may appear clinically as individual behavior or disease (4). Without preparation in pain mechanisms, communication, behavior change, and social context, clinicians may be less prepared to explain why pain can persist or fail to resolve after a targeted intervention.

Expectations are also shaped outside healthcare. Social media and online health communities are common sources of health information, peer support, and treatment stories for people with chronic pain (13, 14). These spaces can validate experiences while exposing people to varied information and opinions about pain management (13, 14). People may therefore enter care with expectations shaped by prior encounters and fragmented information encountered online (13, 14). Pain communication is thus a population level concern, and the National Pain Strategy identifies public education and communication as a core priority (9). Figure 1 summarizes how healthcare systems, education, payment structures, and societal and digital messaging shape chronic pain expectations.

**Figure 1:**
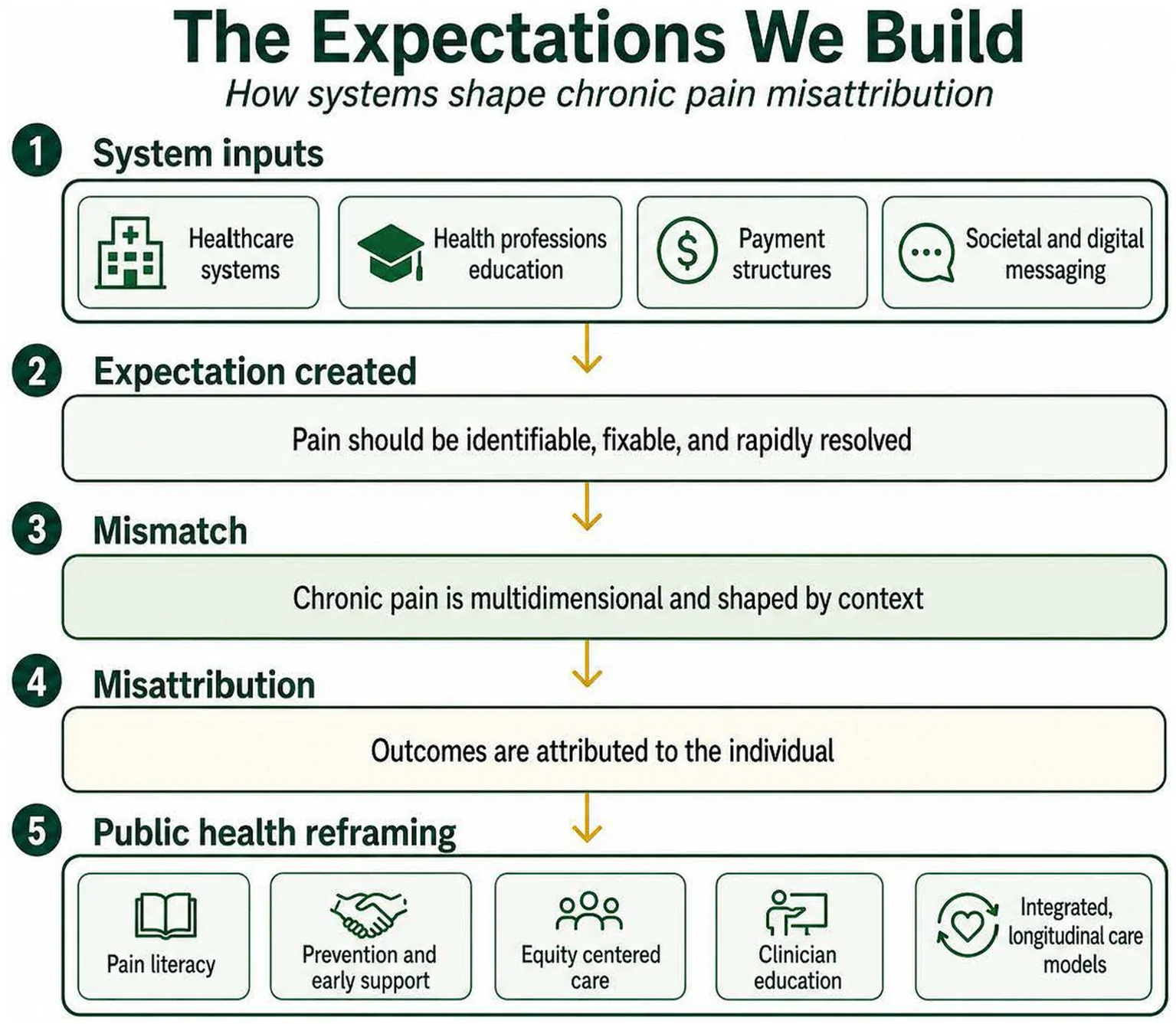
The expectations we build: how systems shape chronic pain misattribution. Healthcare systems, health professions education, payment structures, and societal messaging shape expectations about chronic pain before, during, and after clinical care. These systems may communicate that pain should be identifiable, fixable, and rapidly resolved. Those messages can create expectations that do not align with the complex and nonlinear realities of chronic pain. When chronic pain continues, the mismatch between expectation and reality may lead to frustration, stigma, and misattribution of outcomes to individual factors such as adherence, motivation, resilience, or credibility. A public health reframing shifts attention toward pain literacy, prevention and early support, equity centered care, clinician education, and integrated, longitudinal care models.

## 3 The mismatch between expectations and chronic pain realities

A central challenge is the mismatch between chronic pain and expectations for resolution. Chronic pain persists or recurs for more than 3 months, and ICD-11 recognizes it as primary or secondary to another disease process (15, 16). Chronic pain therefore may not be fully explained by ongoing tissue damage. Yet expectations shaped by acute pain models often assume symptoms should track injury, healing, and recovery. When they do not, the gap can create frustration, uncertainty, and pressure to find a single correctable cause.

The expectation of rapid resolution is reinforced when pharmacological, procedural, or surgical interventions are viewed as the most legitimate pathways to relief. These approaches may be appropriate and beneficial for some patient/clients, but when they become the dominant pathway, they can reinforce a medicalized expectation that chronic pain should have a correctable cause and respond predictably to intervention (2, 17–19). Pain science complicates this assumption. For example, central sensitization describes changes in central nervous system processing that can amplify pain sensitivity and help explain why chronic pain may become disproportionate to identifiable tissue pathology (20). This does not mean that pain is imagined or that structural factors are irrelevant. Rather, chronic pain may reflect dynamic interactions among tissue health, nervous system sensitivity, movement, stress, sleep, beliefs, social context, and prior experiences. When these interactions are not communicated clearly, patient/clients may interpret ongoing pain as evidence that the correct diagnosis or treatment has not yet been found (17, 18).

## 4 From system limitations to individual blame

When expectations surrounding chronic pain care are not met, frustration may be redirected toward the person experiencing pain. Patient/clients whose symptoms continue despite treatment may be perceived as noncompliant, difficult, overly focused on symptoms, or insufficiently motivated (19, 21–23). This response is not just interpersonal but reflects a broader pattern of pain related stigma. Chronic pain is often invisible, difficult to verify through traditional objective testing, and shaped by factors that are not always simple to explain within a biomedical encounter (21–23). Thus, people with chronic pain may experience public, structural, and internalized stigma, including disbelief, dismissal, and delegitimization of their experiences (21). In this context, pain that does not resolve as expected may be viewed as a failure of the person in pain rather than a limitation of the system surrounding the person.

Blame can also move in the opposite direction. Patient/clients may interpret ongoing pain as evidence that the healthcare system or clinician has failed them, particularly when they have been led to expect that the correct diagnosis or intervention should provide relief (17, 18, 22, 23). Epistemic injustice offers a useful lens for this tension. Chronic pain scholarship has shown how patient testimony can be downgraded when clinical expertise and certain forms of evidence are privileged over the person’s account of pain (5, 22, 23). Patient/clients and clinicians may then become positioned against one another even though both are responding to the same expectation of fixability. Instead of examining how care models, communication, reimbursement, and public messaging shape expectations, uncertainty can become personalized as individual failure (5, 21–23).

## 5 Equity implications for underserved populations

The mismatch between expectations and the realities of chronic pain is especially consequential for underserved populations. Chronic pain burden is not evenly distributed across populations. In 2023, chronic pain was more prevalent among women than men, increased as urbanization decreased, and was highest among non-Hispanic American Indian and Alaska Native adults compared with other racial and ethnic groups reported by the National Health Interview Survey (1). Socioeconomic disadvantage is also associated with disabling chronic pain, including among older adults (24). These patterns show that chronic pain outcomes are not only clinical events. Social position, access to resources, geography, and structural conditions shape exposure to pain related risk and access to effective care.

For underserved populations, the mismatch between recommended chronic pain care and real world conditions may be even sharper. Recommended chronic pain care often includes education, active self management, movement, behavioral support, longitudinal follow up, and partnership between the patient/client and clinician (9, 25, 26). Yet these approaches are not consistently available, and when they are recommended, they often assume conditions that many people with chronic pain do not have (27, 28). Care plans may depend on stable insurance coverage, transportation, flexible work schedules, financial resources, health literacy, and access to clinicians who can provide ongoing support (24, 27–29). Social determinants such as income, education, employment conditions, geography, social support, trauma, stigma, and healthcare access are linked to pain prevalence, severity, disability, management, and outcomes (27, 28). When systems do not account for these factors, they may recommend strategies that are evidence informed in theory but difficult to access in practice. Limited progress may then be interpreted as poor adherence or low motivation, when the issue may be a mismatch between recommended care and real world capacity (27, 28).

The expectation that pain should be objectively visible or structurally verifiable can also deepen inequities in clinical encounters (5, 21–23). For patient/clients whose pain is not easily confirmed through imaging, laboratory findings, or observable injury, credibility becomes central to care (5, 22, 23). This is especially concerning for racially and ethnically minoritized patient/clients, whose pain has historically been more likely to be underestimated or undertreated (28, 30). Hoffman and colleagues found that false beliefs about biological differences between Black and White patient/clients were associated with racial bias in pain assessment and treatment recommendations (30). In chronic pain care, diagnosis and treatment often depend on narrative, trust, and interpretation. These biases may increase the likelihood that chronic pain is dismissed, minimized, or attributed to individual behavior rather than understood within a broader structural context.

Structural barriers do more than limit access to care. Structural violence offers a lens for understanding how social and institutional arrangements can intensify harm (6, 31). Among people experiencing chronic pain and social marginalization, pain and care have been shown to be intertwined with stigma, discrimination, trauma, inadequate healthcare, and broader system level inequities (6, 28, 31). Chronic stress has also been linked to physiological processes relevant to chronic pain, and allostatic load may help explain why some people experience greater chronic pain interference and wider pain distribution over time (32, 33). These realities challenge simple explanations that place poor outcomes mainly on individual motivation or resilience. A systems level public health approach must recognize that the capacity to engage in care is not distributed equally. Expectations for self management must be matched by systems that provide realistic support (6, 27, 28, 31).

## 6 Public health implications for recovery, resilience, and systems change

Reframing chronic pain outcomes as system influenced requires moving from recognizing the problem toward specific changes in education, clinical practice, care delivery, and public health policy. A partnership model provides one foundation for this shift. Shared decision making and collaborative care have been identified as promising approaches in pain management, while recent guidance and implementation research emphasize collaborative relationships, active participation, and attention to patient, clinician, interactional, and system level barriers (34–36). Rather than positioning the clinician as responsible for identifying and fixing pain, clinicians and patient/clients can work together to interpret pain, establish realistic expectations, identify barriers to participation, and adjust management strategies over time (34–36). Pain neuroscience education may support this process by helping people reconceptualize pain as less threatening and by addressing psychosocial factors associated with chronic pain (37). However, changing expectations requires more than educating individual patient/clients. The systems that educate clinicians, organize care, reimburse services, and communicate about pain must also change (4, 26, 34–36).

Health professions education offers an early opportunity for action. Existing interprofessional pain competencies provide a foundation for moving pain education beyond knowledge acquisition toward skills that learners can demonstrate in practice (38, 39). Programs can integrate pain science throughout the curriculum rather than limiting it to isolated lectures or courses. Case based learning, simulation, interprofessional experiences, and clinical encounters can require learners to explain chronic pain without relying exclusively on structural pathology, practice shared decision making, identify social barriers to care, and develop multidimensional management plans. Programs can assess these skills through observed clinical encounters, case based examinations, and clinical performance assessments (38, 39). Programs should also consider faculty development to support educators in modeling contemporary pain communication and biopsychosocial clinical reasoning. These approaches move the biopsychosocial model from a concept learners know to a skill clinicians can demonstrate.

Clinicians and patient/clients can also influence expectations within clinical care. Clinicians can discuss expected recovery trajectories, uncertainty, function, and the limitations of structural explanations early in care (34–36). Shared goals can emphasize participation and meaningful activity alongside pain intensity. Patient/clients can identify goals, ask about treatment options, participate in shared decision making, and communicate barriers that affect their ability to engage in a care plan (34–36). Health systems can support this partnership by incorporating patient/client goals, functional outcomes, and barriers to engagement into documentation and quality improvement efforts (26, 35, 36). These strategies shift the focus from whether a patient/client followed a recommendation toward whether the plan was appropriate, accessible, and responsive to the person’s circumstances (5, 26, 34–36).

Care delivery and payment structures require similar attention. Federal recommendations have called for multidisciplinary chronic pain care and improved insurance coverage and payment for pain management services, including care coordination (26). Payers and health systems can support earlier access to evidence informed conservative care, behavioral health services, pain education, interdisciplinary management, and longitudinal support (9, 25, 26). Payment arrangements determine which services are financially supported, while associated quality measures can shape which outcomes are monitored or rewarded. Payers and health systems can align payment arrangements and quality measures to support coordinated, multidisciplinary care and evaluate outcomes such as function, participation, access, and patient/client goals rather than relying primarily on pain intensity or procedure volume (9, 26). Potential advocacy pathways for clinicians and people with chronic pain include professional associations, patient/client advisory councils, health system committees, payer advisory groups, public comment opportunities, and legislative engagement. Professional organizations can strengthen these efforts by developing policy positions and payment recommendations that support accessible, multidisciplinary chronic pain care.

Public health agencies and professional organizations also have a role before chronic pain becomes disabling. Community pain literacy initiatives can distinguish acute from chronic pain, address common misconceptions about imaging and tissue damage, promote active management, and help people identify appropriate pathways to care (9, 26, 29, 35). These messages should account for health literacy, culture, language, accessibility, and the experiences of communities that face greater barriers to care (29). Changing chronic pain outcomes therefore requires more than asking individuals to become more resilient. Educational, clinical, payment, and public health systems must instead create conditions that support recovery, adaptation, and resilience. Table 1 translates these recommendations into specific actions, primary partners, and potential indicators of progress across education, clinical care, payment, advocacy, and public health.

**Table 1:** Translating chronic pain expectations into systems level public health action.

System target	Recommended action	Primary partners	Potential indicators of progress
Health professions education	Integrate pain science, communication, social determinants of health, shared decision making, and multidimensional management throughout curricula. Use case based learning, simulation, interprofessional experiences, and clinical assessment.	Health professions programs, faculty, educational leaders, accrediting and professional organizations	Pain competencies mapped across curricula, learner performance on clinical assessments, faculty development participation
Clinical communication and partnership	Discuss expected recovery, uncertainty, function, patient/client goals, treatment options, and barriers to engagement early and throughout care.	Clinicians, patient/clients, health systems	Documentation of patient/client goals, functional outcomes, shared decisions, and barriers to care
Care delivery	Improve early access to evidence informed conservative care, behavioral health, pain education, interdisciplinary services, and longitudinal support.	Health systems, clinicians, payers, policymakers	Time to appropriate care, access to multidisciplinary services, continuity of care, functional outcomes
Payment structures	Support reimbursement for education, care coordination, behavioral health, interdisciplinary care, and other services that support long term chronic pain management.	Public and private payers, health systems, professional organizations, policymakers	Coverage policies, reimbursement for coordinated services, reduced access barriers
Advocacy	Create pathways for clinicians and people with chronic pain to influence institutional and public policy through advisory councils, professional organizations, payer engagement, public comment, and legislative advocacy.	Patient/clients, clinicians, advocacy groups, professional organizations	Patient/client representation in decision making, policy proposals, institutional or payer changes
Public pain literacy and equity	Develop accessible community education that distinguishes acute from chronic pain, addresses common misconceptions, promotes active management, and accounts for health literacy and structural barriers.	Public health agencies, community organizations, professional organizations, health systems	Reach of educational efforts, pain knowledge, accessibility across populations, disparities in access and engagement

This table identifies recommended actions, primary partners, and potential indicators of progress across health professions education, clinical care, payment, advocacy, and public health.

## 7 Discussion

Chronic pain outcomes are often interpreted through the lens of individual responsibility, but this framing is incomplete. Expectations surrounding chronic pain are built across healthcare systems, health professions education, payment structures, public messaging, and social conditions (4, 9, 12, 26–28). When these expectations emphasize rapid diagnosis, passive treatment, and definitive resolution, they do not align with the complex and often nonlinear realities of chronic pain (17–20). The consequences of this mismatch are not distributed evenly. They are amplified for populations already navigating structural barriers, inequitable access, and higher burdens of chronic pain (1, 24, 27, 28, 30). Taken together, these perspectives locate chronic pain outcomes at the intersection of embodied experience, clinical knowledge, and structural conditions rather than within any one level alone (2–6).

Improving outcomes requires more than asking patient/clients to be more adherent, motivated, or resilient. This reframing is not intended to transfer blame from patient/clients to individual clinicians. It makes visible the conditions that shape the choices, expectations, and constraints experienced by both. It requires systems that create more accurate expectations, provide realistic support, and align care with the lived realities of chronic pain. This shift is both clinical and public health oriented. It calls for better communication in care encounters, stronger pain education in health professions training, broader pain literacy in communities, and policies that make prevention, early support, and long term care more accessible (12, 26, 28, 29, 34).

This Perspective should be interpreted in light of its the purpose and scope of the article. The manuscript does not present primary data or a systematic review. Instead, it uses selected public health, pain science, education, and equity literature to advance a systems level argument about how expectations are built and how misattribution can occur. Future research should move beyond identifying these problems toward testing strategies for change. This includes evaluating pain education models in health professions curricula, approaches to improving pain literacy in communities, models of shared decision making and longitudinal care, and payment reforms that improve access to multidisciplinary services. Implementation should also be evaluated across populations to determine whether these approaches reduce rather than reproduce existing inequities. Chronic pain outcomes should not be separated from the systems that shape expectations, access, recovery, and resilience.

## Data Availability

The original contributions presented in the study are included in the article/supplementary material, further inquiries can be directed to the corresponding author.
